# *Alona iheringula* Sinev & Kotov, 2004 (Crustacea, Anomopoda, Chydoridae, Aloninae): Life Cycle and DNA Barcode with Implications for the Taxonomy of the Aloninae Subfamily

**DOI:** 10.1371/journal.pone.0097050

**Published:** 2014-05-30

**Authors:** Erika dos Santos Silva, Cínthia Bruno de Abreu, Tereza Cristina Orlando, Célio Wisniewski, Maria José dos Santos-Wisniewski

**Affiliations:** 1 Instituto de Ciências da Natureza, Universidade Federal de Alfenas (UNIFAL-MG), Gabriel Monteiro da Silva, 700, Alfenas, Minas Gerais, Brazil; 2 Instituto de Ciências Exatas, Universidade Federal de Alfenas (UNIFAL-MG), Gabriel Monteiro da Silva, 700, Alfenas, Minas Gerais, Brazil; Catalan Institute for Water Research (ICRA), Spain

## Abstract

Knowledge of reproductive rates and life cycle of the Cladocera species is essential for population dynamic studies, secondary production and food webs, as well as the management and preservation of aquatic ecosystems. The present study aimed to understand the life cycle and growth of *Alona iheringula* Kotov & Sinev, 2004 (Crustacea, Anomopoda, Chydoridae), a Neotropical species, as well as its DNA barcoding, providing new information on the Aloninae taxonomy. The specimens were collected in the dammed portion of the Cabo Verde River (21°26′05″ S and 46°10′57″ W), in the Furnas Reservoir, Minas Gerais State, Brazil. Forty neonates were observed individually two or three times a day under controlled temperature (25±1°C), photoperiod (12 h light/12 h dark) and feeding (*Pseudokirchneriella subcapitata* at a concentration of 10^5^ cells.mL^−1^ and a mixed suspension of yeast and fish feed in equal proportion). Individual body growth was measured daily under optical microscope using a micrometric grid and 40× magnification. The species had a mean size of 413(±29) µm, a maximum size of 510 µm and reached maturity at 3.24(±0.69) days of age. Mean fecundity was 2 eggs per female per brood and the mean number of eggs produced per female during the entire life cycle was 47.6(±6.3) eggs per female. The embryonic development time was 1.79(±0.23) days and the maximum longevity was 54 days. The species had eight instars throughout its life cycle and four instars between neonate and primipara stage. The present study using molecular data (a 461 bp smaller COI fragment) demonstrated a deep divergence in the Aloninae subfamily.

## Introduction

Cladocera are important for energy transference in the food chain in natural ecosystems. These organisms are used as a food source for larvae and juvenile fish in aquaculture due to their high nutritional value and short development time [1], [2].

The family Chydoridae Dybowski & Grochowski, 1894 emend. Frey, 1967 inhabit the littoral region of water bodies and normally lives associated with macrophytes, periphyton or sediment, and are represented by substrate scraper organisms [3], [4], [5]. The littoral region plays an important role in productivity and nutrient cycling in aquatic ecosystems. Environments colonized by macrophytes have high environmental heterogeneity because they have a high diversity of ecological niches and species biodiversity [6].

Studies on the reproductive rates and life cycle of Cladocera species allow a better understanding of the role each plays in zooplankton communities. They also provide data for other studies, such as secondary production determination and toxicity tests, and contribute to many ecological studies, such as energy flow, population dynamics and functional groups [7], [8], [9].

Cladocera reproduce mainly by parthenogenesis, but under unfavorable conditions, the birth of males and production of resting eggs may occur, which contribute to the spread and propagation of these species [10], [11]. Males are little known or unusual in some species of Cladocera and probably associated with specific environmental changes. These changes can modify the development rates and the life cycle of species of Cladocera [12], [13].

Some studies have been carried out with a focus on the life cycle of parthenogenetic females of the species of the chydorids [14], [15], [16], [17], [18], [19], [20], [21]. In Brazil, the life cycle of C*hydorus dentifer* and *Acroperus harpae* was analysed by Melão [13], *Chydorus pubescens by* Santos-Wisniewski et al [22] and *Coronatella rectangula* by Viti et al [23].

Due to the high richness of the chydorids found in freshwater, the number of species whose life cycle has been studied is still very small, compared to other families of Cladocera, covering approximately 18.31% of the known species. Therefore, it is necessary to extend such studies to a larger number of species.

*Alona iheringula* Kotov & Sinev, 2004 belongs to the Aloninae subfamily. It is a Neotropical species and belongs to the *Alona costata* Sars, 1862 [24], species complex. It was considered as a synonym of *Alona rustica* Scott, 1895 [25] for some time, but Sinev [26] showed clear differences and considered it to be *Alona iheringi* Sars, 1901. Later, it was renamed as *Alona iheringula* by Kotov and Sinev [24].

The region known as the DNA barcode is based on a segment of the mitochondrial gene cytochrome c oxidase I subunit (COI) and has demonstrated to be an important molecular marker in taxonomic classification [27].

For the Subphylum Crustacea and especially for the Chydoridae family, the COI region was demonstrated to be effective in separating taxa [28], [29] and detecting crustaceans as prey in ecological studies [30] and cladocera aquatic invasions [31]. Important COI studies were made for *Simocephalus* [32], *Eurycercus* [33], *Leberis chihuahuensis* from the northern Mexican semi–desert [34]. Studies using Branchiopoda species diversity in Canada [35] and Cladocera species from Guatemala and Mexico [36] have highlighted the DNA barcode as a useful tool for many applications. We sequenced the COI from *A. iheringula* with the aim of clarifying its specific status and taxonomic position.

## Materials and Methods

Some females were collected in the littoral region of a dammed portion of the Cabo Verde River/Furnas Reservoir (21° 26′05″ S, 46° 10′57″ W), in the southern region of Minas Gerais State, Brazil, by vertical and horizontal hauls using a zooplankton net of 68 µm mesh size. It is a public area and no special permission is necessary to authorize access. The species involved are not endangered or protected. Therefore, there are no restrictions on collecting. The sample was collected near macrophytes in February 2009.

**Figure 1:**
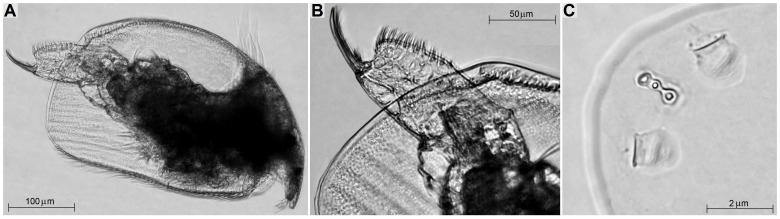
*Alona iheringula* (Crustacea: Anomopoda; Chydoridae): A - general view; B - post-abdomen; C - head pores.

In the laboratory, parthenogenetic females of *Alona iheringula* (Fig 1A–C) were isolated and placed in 2 L beakers containing reconstituted water, according to ABNT [37]. The culture media and food suspension were renewed every two days. Specimens were acclimated for about 10 generations (30 days). The pH of the culture media was 7.4; the electric conductivity was 180 µS.cm^−1^ and the hardness was 46 mg.L^−1^ CaCO_3_. Experimental cultures were maintained in germination chambers with a photoperiod of 12h-light/12h-dark and temperature of 25(±1)°C (a mean temperature observed in the sampling area when the organisms were collected). The organisms were fed with *Pseudokirchneriella subcapitata* algae, cultured in Chu 12 medium and cropped in the exponential phase, with 10^5^ cells per individual and a suspension of mixed suspension (fish-food and biological yeast), supplied in equal proportions [37], [38].

Twenty females were isolated and maintained until the production of neonates. The 40 neonates of less than 24 hours old were placed in 100 mL of culture medium in polypropylene bottles and kept in a germination chamber with temperature, light and feeding conditions as specified above. The isolated organisms were observed two or three times a day under the stereoscopic microscope to obtain life cycle data (development times, longevity and fecundity). Individual body growth was measured daily under optical microscope using a micrometric grid and 40× magnification.

In the same collecting coordinates, qualitative samples of the zooplankton community were collected (near the macrophytes) using a net of 68 µm mesh size, every two months, from June 2008 to October 2009. The samples were fixed in formalin 4% and kept in the Coleção do Laboratório de Limnologia (sample CL3053), at the Universidade Federal de Alfenas, MG (UNIFAL-MG). From Secchi disk and chlorophyll-*a* data, obtained from the same place, the Trophic State Index was calculated by Carlson methodology, adapted by Toledo et al [39]. The chlorophyll concentration was determined using the extraction method with 90% acetone as described in Golterman et al [40].

For the DNA barcode analysis, the specimens were fixed with 90% EtOH and placed into pure water for 12 h for cleaning. Genomic DNA was extracted using phenol extraction and ethanol precipitation [41]. To amplify the mitochondrial COI gene, the internal primers Chy-f 5′-TTG GGG ATG ATC AAATTT ATA ATG T-3′ and Chy-r 5′-AGA GGT ATT CAG ATT TCG ATC TGT CA-3′ [42] were used. PCR reactions had a total volume of 25 µL and were performed according to Ivanova et al [43] using Platinum *Taq* (Invitrogen) as the enzyme. The PCR conditions were 94°C for 3 min as initial denaturation and 40 cycles of 94°C for 30 s, 50°C for 30 s and 72°C for 90 s. Direct DNA sequencing was done using purified Exo-SAP (Fermentas) PCR products, carried out in a *3130xl Genetic Analyzer* (Life Technologies, Carlsbad/CA/USA) automated DNA sequencer, following the manufacturer's instructions. The sequences were obtained bidirectionally for accurate reading (Genbank access number KF383284).

The *Alona iheringula* COI smaller fragment (using Chy-f and Chy-r, [42]) was aligned in MEGA 5 [44] with other Aloninae COI sequences obtained from Barcode of Life Data Systems (BOLD) (http://www.barcodinglife.org, [45]). The Kimura 2-Parameter (K2P) distance model was used to calculate sequence divergences and the GTR+G+I was found to be the best substitution model obtained for the alignment (+*G*, parameter  =  2.7514;[+*I*], 60.4423% sites). Identification trees were generated by MEGA 5 [44] facilities. Nonparametric bootstrapping was performed using 1000 replicates.

## Results

### *Alona iheringula* culture and life cycle

The sampling area in the dammed portion of the Cabo Verde River was characterized as a shallow (3–9 m depth) mesotrophic environment (Trophic State Index of 47), with macrophytes (Myriophylum aquaticum, Eichornia azurea, Typha domingensis and Pistia stratiotes). The water had a mean pH of 5.2 (±0.6) (slightly acidic), temperature ranged from 20 to 25°C, dissolved oxygen concentration from 4.5 to 8.0 mgL^−1^ and low medium conductivity, 41.7 (±9.5) µS.cm^−1^.

**Figure 2:**
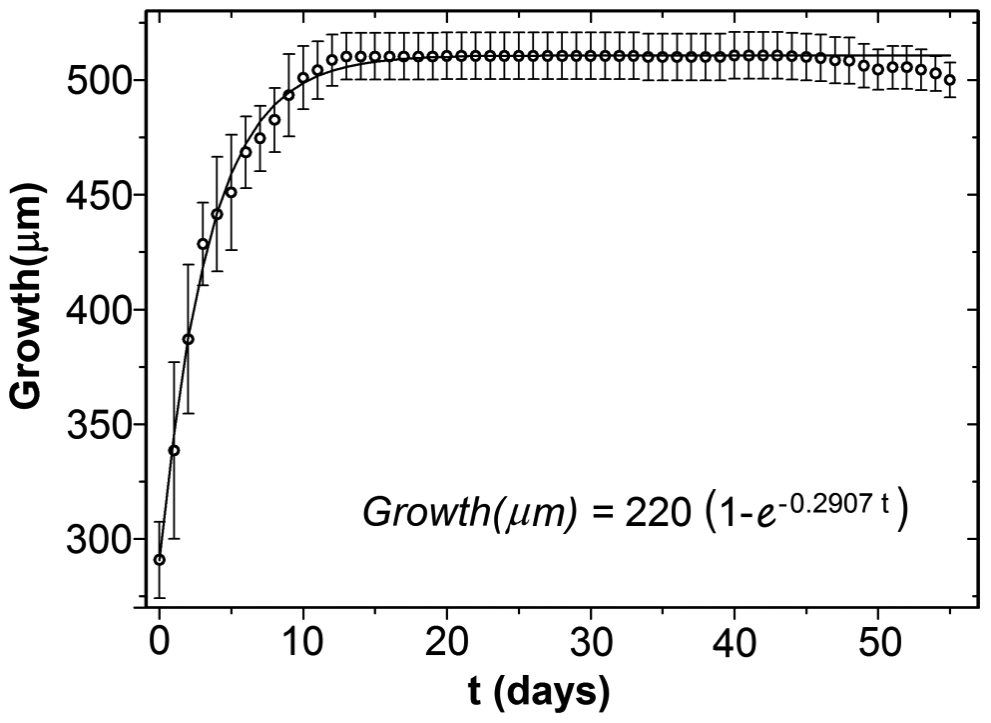
Mean individual growth curve of *Alona iheringula* (Cladocera Chydoridae) grown in the laboratory at 25°C.

The neonate had an average size of 288 (±20) µm, reaching maturity after 3.3 (±0.7) days and 413 (±29) µm in mean size. The maximum size (510 µm) was reached in 11 days. The mean individual growth curve of *Alona iheringula* is shown in figure 2.

The main results for the life cycle of *Alona iheringula* are presented in Table 1. This species has a maximum longevity of 54 days and mean longevity of 46 (±6) days. The mean of embryonic development time was 1.79 (±0.24) days. The mean fecundity of parthenogenetic females was 2.0 eggs per female per brood, producing a mean of 47.6 (±6.3) eggs per female during the entire life cycle. The species had eight instars throughout its life cycle and four instars from neonate to primipara stage.

**Table 1:** Life cycle data for *A. iheringula* (Cladocera, Aloninae) from laboratory cultures at 25(±1)°C.

Mean neonate size	288 (±19) µm
Mean primipara size	413 (±29) µm
Minimum primipara size	360 µm
Maximum adult size	510 µm
Mean egg number (whole cycle)	47.6 (±6.3) eggs per female
Mean fecundity	2.0 eggs per female per brood
Maximum longevity	54 days
Mean longevity	46 (±6) days
Mean embryonic development time	1.79 (±0.23) days
Primipara age	3.24 (±0.69) days
Instar number (between neonate and primipara)	3
Maximum instar number (whole life cycle)	8

From qualitative sample analysis, during the collecting period, A. iheringula was only registered in February, August, and October 2009, in a sample collected near macrophytes. Chydoridae and Daphnidae families were the most representative, with 42% and 41% of total species. Bosminidae, Sididae and Ilyocryptidae families had a lower representation, with 11%, 3% and 2% of total species found.

### COI analysis

The *Alona iheringula* COI sequence obtained using the Chy-f and Chy-r primers [42] was 461 bp long. The base composition for *Alona iheringula* COI sequence was: T = 39.5%; C = 17.57%; A = 23.0%; G = 19.96%. The calculated A–T content was 62.5%.

As this size using the Chy-f and Chy-r primers [42] is smaller than those usually obtained with the universal primers HCO2198 and LCO1490 of 658pb [46], a test using all COI sequences for *Alona* and *Leberis* species found at BOLD (http://www.barcodinglife.org) was performed using the 658 bp and the 461 bp smaller fragment corresponding to the aforementioned isolated region for *A. inheringula*, in order to verify if a smaller COI sequence could modify the taxonomic status. The identification tree obtained using both the 658 bp and the 461 bp fragments provided similar results (data not shown), not changing the taxonomic status among all the 14 sequences (not including the *A. iheringula* sequence).

**Figure 3:**
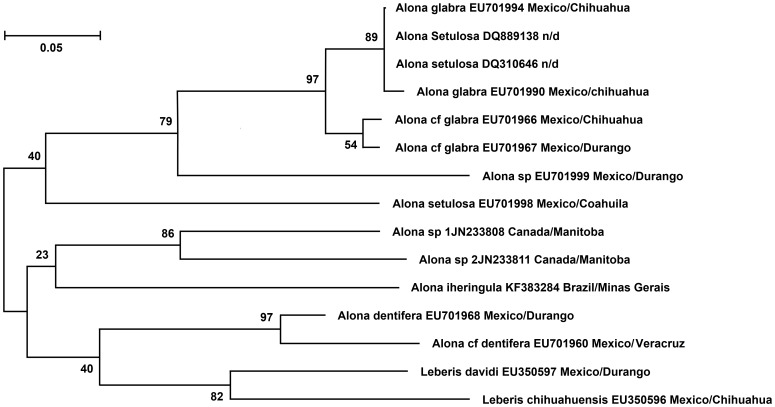
Identification COI tree of *Alona iheringula* and other *Alona* and *Leberis* species using a 461bp-COI fragment inferred by ML. Tree with the highest log likelihood (-2487.9868) is shown. Numbers in each node designate percentage of bootstrap support (for 1000 replicates). The bar shows the number of substitutions per site. Genbank access number and locality are located after each species name. n/d: not determined.

A genetic divergence ranging from 18.5% to 23.1% among *Alona iheringula* (GenBank access number KF383284) and other species of the genus *Alona* was found (Table 2). Among the 15 COI analyzed sequences, the identification tree (Fig. 3) shows *Alona iheringula* as closely related to: *Alona* sp1 (JN233808) and *Alona* sp2 (JN233811), both from Manitoba, Canada. *A. glabra* (EU701990 and EU701994, from Chihuahua/Mexico) and *A. setulosa* (DQ889138 and DQ310646, with undetermined location) were mixed together as a well supported group exhibiting an 89% bootstrap value. However, *A*. cf *glabra* (EU701966 and EU701967) separate as another clade, also with the *Alona* sp (EU701999) as a separate clade (Fig. 3). *A. dentifera* and *A*. cf *dentifera* cluster together with the two *Leberis* species with a poorly supported bootstrap value of 40% (Fig. 3).

**Table 2:** K2P Genetic distance between COI sequences of *A. iheringula* and other *Alona* and *Leberis* species. Genbank access numbers are located after each species name.

	1	2	3	4	5	6	7	8	9	10	11	12	13	14
1. *A. iheringula* (KF383284)														
2. *A. sp* (EU701999)	23.1													
3. *A. sp 1* (JN233808)	20.2	21.0												
4. *A. sp 2* (JN233811)	21.4	20.4	16.4											
5. *A. dentifera* (EU701968)	18.5	20.4	19.0	20.7										
6. *A. cf dentifera* (EU701960)	19.0	21.3	19.9	21.5	8.1									
7. *A. glabra* (EU701990)	21.1	16.8	21.3	21.3	20.7	21.5								
8. *A. cf glabra* (EU701966)	21.0	16.0	20.1	21.6	20.4	21.5	5.9							
9. *A. cf glabra* (EU701967)	20.7	15.4	21.1	21.9	19.5	21.0	6.2	1.8						
10. *A. glabra* (EU701994)	21.1	17.1	22.0	21.9	21.3	21.5	1.1	5.2	5.0					
11. *A. setulosa* (DQ889138)	21.1	17.1	22.0	21.9	21.3	21.5	1.1	5.2	5.0	0.0				
12. *A. setulosa* (DQ310646)	21.1	17.1	22.0	21.9	21.3	21.5	1.1	5.2	5.0	0.0	0.0			
13. *A. setulosa* (DQ701998)	20.7	19.3	21.3	22.7	18.2	20.2	19.4	20.5	19.9	19.9	19.9	19.9		
14. *Leberis davidi* (EU350597)	20.0	22.4	21.0	19.6	17.1	18.2	19.3	20.2	19.6	19.0	19.0	19.0	20.4	
15. *L.chihuahuensis* (EU350596)	22.3	20.9	23.4	20.2	18.3	17.6	19.9	19.9	19.3	19.9	19.9	19.9	19.3	15.4

## Discussion

In Brazil, there are records of occurrence of *A. iheringula* in Pará, Mato Grosso do Sul, Maranhão, Goiás, Distrito Federal, Minas Gerais, Rio de Janeiro, São Paulo and Rio Grande do Sul states [47], [5], [48], [49]. In Minas Gerais state, it was recorded in the Rio Doce Basin, San Francisco Basin [50], [51] and in a dammed portion of the Cabo Verde River/Furnas Reservoir (present study). The sampling area had a slightly acidic pH. Tolerance to acidic environments is a typical physiological adaptation of the *Alona rustica* group, which is the closest to *A. iheringula,* and the occurrence of *A. rustica* Palearctic is known in acid systems [52]. The mean water temperature was close to that used in the experiment (25±1°C) and had good oxygenation and low electrical conductivity values compared to other reservoirs [53], [54], [55].

The mean size of the *A. iheringula* neonate (288±19 µm) was lower than that found by Melão [13] for *Chydorus dentifer* (302 µm) and by Sharma and Sharma [20] for *Alonella excisa* (368 µm). According to Kotov [19], some species have a special molt, immediately after being released from the mother's pouch, corresponding to the first juvenile stage. The first measurement of this study probably corresponds to this stage. The maximum size reached by the *A. iheringula* (510 µm) was similar to that found by other authors and species of the subfamily Aloninae. Kotov and Sinev [24] recorded a mean size from 380 to 450 µm for parthenogenetic females of *A. iheringula,* in São Paulo State. Sinev [56] found a mean size of 500 µm for *Alona costata*, in a European study.

Temperature influences the species development, when maintained in laboratory under non-limiting food conditions, and there is a negative relationship between temperature and development time and longevity [57], [58], [7]. The mean longevity of *A. iheringula* (46±6 days) was similar to that obtained for *Leydigia ciliata* at temperatures of 28 and 30°C [18]. Martínez-Jerónimo and Gómez-Díaz [21] also found differences in development times associated with temperature for *Leydigia louisi mexicana* species. Bottrell [15] found 74 days at 10°C for *Acroperus harpae*, showing the temperature dependency for development time and longevity.

The quality, diversity and quantity of food also influences longevity [59], [58]. Melão [13] found shorter longevity for *Acroperus harpae* than in the present study, using only algae as food, in otherwise similar conditions. Although food was offered *ad libitum*, the less diverse food offered probably influenced longevity, which decreased.

According to Smirnov [3], the duration of the life cycle of the chydorid species ranges from 5 to 94 days and, according to Lynch [60], from 24 to 42 days. Therefore, the maximum longevity recorded for *A. iheringula* is within the range established by Smirnov [3].

The age of the primipara of *A. iheringula* (3.24±0.69 days) was similar to that found for *Leydigia acanthocercoides* (3 days) at temperatures of 28 to 30°C [16] and *Alonella excisa* (3.17 days) at 19 and 23°C [20]. Santos-Wisniewski et al [22] recorded the primipara age of 2.4 days for *Chydorus pubescens*, which is lower than registered for *A. iheringula*.

The embryonic development time (1.79±0.24 days) was close to that found by other authors for some Chydoridae species [13], [22] at the same temperature. Embryonic development depends on several factors. The influence of temperature and quality of food in embryonic development of the Cladocera species has been observed by several authors [61], [58], [7], [22], [21]. Embryonic development times may also be related to egg sizes from species of Cladocera [13], [62]. The species in this study belongs to the subfamily Aloninae, which has characteristics such as a more elongated body, larger eggs and longer embryonic development time than species that belong to the subfamily Chydorinae, which has a small body, small eggs and short development time [22].

The mean fecundity (2 eggs per female per brood) and total fecundity (47.6±6.3 eggs per female) were similar to that found by other authors [16], [18], [20], [22], [21] for other species in the Chydoridae family. This feature may be less variable among species. According to Kotov [63], the Chydoridae family has a laterally compressed body, which is a morphological adaptation to benthic environments. Other families like Daphnidae, Moinidae and Sididae, for example, have rounded bodies, which are adapted to living free in a limnetic environment. Therefore, different body shapes of Cladocera species are likely to influence the number of eggs produced per brood, since their flattened bodies have a smaller incubator chamber and thus reduce the number of eggs produced.

Four juvenile stages were recorded for *A. iheringula.* According to Bottrell [15] the number of instars ranges from 3 to 8 stages for the Cladocera species. The number is generally constant for all species, although it may increase under limited food conditions [18], [19], [20]. Smirnov [3], Lynch [60] and Venkataraman [18] registered three instars for Chydoridae species and showed that the range is lower than observed for other Cladocera families, which may have anywhere from 2 to 7 juvenile stages. For adult stages, *A. iheringula* presented 8 stages, fewer than those found by other authors [3], [16], probably because this is an intrinsic characteristic of this species.

A. *iheringula* is characteristic of the littoral region, where it is associated with aquatic macrophytes, and consequently it was only recorded in qualitative samples collected near macrophytes. The presence of macrophytes provides greater species diversity because they provide shelter and a food source for many animal species and produce high environmental heterogeneity [64].

Collections in the macrophyte region need to be intensified. On the days that *A*. *iheringula* was found, there was a high abundance of macrophytes (*Myriophylum aquaticum*, *Eichornia azurea*, *Typha domingensis* and *Pistia stratiotes*) nearby and greater frequency and representativeness of Chydoridae species.

The knowledge of the life cycle and laboratory culture facilitates the molecular studies of the Cladocera species as a large number of specimens are derived from the same clonal population, which is very interesting as a large amount of DNA can be obtained for different molecular studies.

This study provides new information regarding the subfamily Aloninae, stressing that it is a group whose taxonomy is complex and still not very well defined. In recent years several species of the subfamily Aloninae have been described and redescribed [65], emphasizing the need for taxonomic revision of the group, which is underestimated.

The percentage found for A-T (62.5%) is similar to the 60% A-T percentage for COI of Chydoridae [42], [28].

As shown in the identification tree (Fig. 3) the classification and taxonomy can be obtained with a smaller fragment of COI (461 bp), amplified with Chy-f and Chy-r [42], given the difficulty of amplifying this species with universal primers [46], thus showing that this smaller sequence is sufficient for molecular taxonomy for the Cladocera species analyzed.

The determination of the *A. iheringula* COI added new information to daunting challenge of elucidating the still unclear taxonomy of the Aloninae subfamily. Here we found a strong genetic differentiation between the alonine clades. The present study using molecular data (a smaller COI fragment of 461 bp) also corroborates the polyphyletic nature of the Aloninae group as already discussed using morphological [66] and molecular data (using COI, 18S rRNA and 16S rRNA, [28]).

The use of molecular depth studies as the present one and morphological characters with a large number of species from different localities can resolve some problems of taxonomic issues and further establish the most probable phylogeny of the Aloninae subfamily. This will help better establish taxonomic status for the already described and the new morphospecies, as important aspects for detection of distinct possible ecological niches and different needs for preservation studies on plankton biodiversity and biotechnological purposes, including determination of new model species for biomonitoring experiments and xenobiotic degradation pathways. Also, efforts should be directed towards the growth of global databases such as BOLD and Genbank, in order to help future identifications and phylogenetic studies.
